# Acute Synovitis, or Open Joint

**Published:** 1897-03

**Authors:** W. J. Martin

**Affiliations:** Kankakee, Ill.


### ACUTE SYNOVITIS, OR OPEN JOINT.

By W. J. Martin, V.S.,
KANKAKEE, ILL.

Open joint is a lesion often met with in veterinary practice, and is one which causes the veterinary practitioner much anxiety and hard work in his endeavor to restore the animal to its former condition of soundness, or, at least, to a condition in which it can perform a certain amount of hard labor for its owner. Very often all this medical skill is of no avail, and what seemed to the layman a trivial injury speedily ends in the death of the animal or a hopelessly crippled condition.

*Etiology.* Open joint may occur in any joint of the body, though the stifle, knee, hock, and navicular joints are most frequently affected. Very often the navicular joint is opened either directly by a penetrating nail or by suppuration from the inflammation resulting from such an injury. Open joint may also follow a severe attack of acute articular arthritis. The most prolific causes of open joint, however, are traumatic injuries, such as kicks upon the joint by another animal, falling upon the knees while drawing heavy loads, or, in driving and race horses, while being driven at a high rate of speed. The pitchfork in the hands of an angry groom is often a prime cause of this disease.

*Semeiology.* Very often the wound of the joint appears to the casual observer of a very trivial nature, so much so that perhaps a couple of days may pass without the animal showing much pain or stiffness in the injured limb. At about this time, however, the animal does begin to show signs of being stiff in the injured joint, and also appears unwilling to advance the limb forward, if made to walk, or to rest it firmly upon the ground if at rest. In other cases the skin and even the deep tissues, such as the tendons which pass over the joint, and even the capsular ligament which surrounds the joint, will be found lacerated and destroyed, and synovial fluid will be seen escaping directly from the joint. It is well in all cases of open joint to be able to discriminate between the mere opening of a synovial bursa, a synovial sheath, and that involving the true articulation of a joint. The former of these is often of a trivial character and readily yields to proper treatment. The second is of a more serious nature, and often gives rise to the destruction of the fibres of the tendons which pass through them. An incised wound caused by a sharp-cutting instrument is much more amenable to treatment than one caused by a blunt one. The latter often bruises and lacerates in a most serious manner, and, if the joint is not directly opened by the injury, it is very apt to become so in a few days, owing to the sloughing of the bruised parts.

A horse suffering from open joint in the early stage presents many typical symptoms which the eye of the experienced practitioner readily detects. A horse has received an injury, say, on the hock-joint by a kick from another horse. A few drops of blood may be seen on either the inner or outer surface of the joint, as the case may be. The wound is to all appearance trifling, and the owner keeps the animal at work. By the second day, however, the joint is found to be somewhat swollen and stiff, the animal shows much pain and uneasiness upon being compelled to move around in the stall; at this time also there may be seen a slight oozing out of fluid at the point of injury; this fluid is of a clear, oily nature and very slippery, having a tendency to glide rapidly over the hairs below the seat of injury without adhering to them. This is synovial fluid. We are then confronted by a case of true open joint, together with a long line of pathological evils that arise from it.

Very often in the first stage of the injury the synovial fluid may be mixed with a whitish or frothy-looking serum. In the later stages—that is, after the injury is of several days' duration—this discharge often assumes a bloody, purulent form, in which more or less pieces of the cast-off edges of the diseased synovial membrane and the articular cartilage will be found. At this stage the whole joint is greatly swollen and intensely painful, owing to the exudation of lymph into the surrounding tissue, which becomes partly organized. If the disease has been going on for, say, ten days, we find the lateral portion of the joint the seat of numerous fistulous tracts leading from within outward from the bones of the joint.

This description perhaps ought only to apply to joints which are composed of numerous bones, such as the hock, knee, etc., because the more bones there are in the articulation the greater will be the amount of inflammation present.

In nearly all cases of open joint the appetite will be impaired, while thirst will be much increased. The temperature will often be found to rise from three to six degrees above normal. The pain always is most intense, the animal frequently catching the limb up in a spasmodic manner nearly to the body, and then gradually lowering it until the weight of the limb is supported upon the toe of the hoof. The breathing is accelerated, respiration often being from thirty to thirty-five per minute; perspiration bedews the flanks and caput muscles of the shoulder; the face presents a pinched and anxious expression, plainly showing the great amount of pain the animal is suffering. In a well-bred animal, and if of a highly nervous temperament, all these symptoms mentioned may be much more marked. On the other hand, if the animal is of a dull, phlegmatic disposition, but little constitutional disturbance may be present.

*Termination.* As termination of this disease we may have resolution, suppuration, gangrene, articular arthritis, and anchylosis. In severe cases of open joint occurring in the horse resolution takes place but seldom; it is much more apt to end in the destruction of the articular cartilage covering the ends of the bones, and even the vascular structures of the bones themselves are laid bare by the inflammation. As a result of this inflammation we have an exudation of bone-forming lymph taking place within the cancelli and Haversian canals of the bones forming the articulation, which effectually fill up these openings, and becoming confluent become organized into bone, thus binding the entire articulation into a solid mass of bone. This pathological condition is the most common termination of open joint. Frequently the diseased joint will become enormously enlarged by the osseous deposits which take place around it.

*Treatment.* There seems to be a wide difference of opinion among veterinarians the world over in regard to the proper treatment of open joint. Some men who are considered authorities on the practice of veterinary medicine and surgery advocate a certain line of treatment which might be considered diametrically opposite to that advocated by others equally prominent. On account of this difference of opinion the careful practitioner must largely rely upon his own resources, and base his treatment largely upon common sense and the facts which each particular case presents to his observation. In cases where there is much laceration of the skin and tissue covering a joint, if, after removing all foreign bodies which may be in the wound, such as small gravel, pieces of wood, dirt, etc., it is then found impossible to bring the edges of the wound together with sutures, and the injury has but just happened, I try to keep the animal as quiet as possible, and, at the same time, to exclude atmospheric air and disease-germs. I brush lightly over the wound a solution of flexible collodion. In the knee, if the seat of injury is between the radius and the upper row of carpal bones, I find it an excellent plan to render flexion of the limb impossible. This may be accomplished either by means of a splint applied to the posterior part of the leg, or by means of a plaster-of-Paris bandage placed around the joint, leaving the seat of injury exposed for dressing. This cannot be done so readily in the hock-joint, though even here it has given me most excellent results.

All probing and unnecessary handling of the injured parts are to be avoided. The incautious use of the probe has often changed what at first was a deep wound, but not involving the joint, into that of an open joint by the irritation set up by the probe.

Where there is much inflammation present in the joint, together with the discharge of bloody pus mixed with synovia, showing that destructive tissue-metamorphosis is going on within the joint, warm anodyne applications must be used. These may be in the liquid form, though I prefer them in the form of a cataplasm of linseed-meal medicated with some antiseptic, such as phenic acid, tincture of iodine, or oil of eucalyptus. Very often, in the early stages of the disease and where the opening into the joint is small and not much laceration of external tissue has taken place, a smart blister applied all around the joint serves effectually to stop the flow of the synovia. Warm water in which a small amount of chloride of zinc has been dissolved, say, one drachm to a quart of water, may also be allowed to flow in a gentle stream over the wounded joint. This serves to mechanically coagulate the synovial fluid, which in turn acts as a plug, thereby arresting the flow of the synovia. In no case should this plug be removed or in any way disturbed in dressing the joint.

In those cases of some days' standing, and when the discharge is composed largely of pus, it will be useless to attempt to arrest the discharge from the joint by any of the means mentioned here, because, even if we did succeed in arresting the flow at the seat of injury, it would soon break out again in some other part of the joint. At this stage of the disease recovery by anchylosis is our only hope.

Should the body-temperature run high, antipyretics, such as quinine, salicylate of sodium, acetanilid, etc., should be given internally. If the bowels become constipated, which they are very apt to be, enemas of warm water should be given, followed by a saline laxative. Saline laxatives are indicated here because they have really a double action—acting upon the urinary organs, and thereby increasing their activity and thus eliminating from the blood its morbid products.

During the first few days, while the temperature is high, the animal if inclined to eat should be fed most sparingly; bran and a small amount of hay is all the animal should receive.

At the beginning of the disease the animal should be placed in the slings. These should not be drawn too tight at first, because if the animal is raised too high he will probably worry very much, and the sling will then do more harm than good. When the flow of synovia has ceased and the joint remains hot and swollen, blisters should be applied around the entire joint; later, if the process of anchylosis is taking place slowly, these should be of the most potent kind to overcome the existing lameness. The best blister for this purpose is the ointment of biniodide of mercury and cantharides in combination, two parts of the former to one of the latter. When these fail resort must be had to the actual cautery, and this must be used with no light hand.

